# Extraction of Surface-Related Features in a Recurrent Model of V1-V2 Interactions

**DOI:** 10.1371/journal.pone.0005909

**Published:** 2009-06-15

**Authors:** Ulrich Weidenbacher, Heiko Neumann

**Affiliations:** Institute of Neural Information Processing, University of Ulm, Ulm, Germany; Tel Aviv University, Israel

## Abstract

**Background:**

Humans can effortlessly segment surfaces and objects from two-dimensional (2D) images that are projections of the 3D world. The projection from 3D to 2D leads partially to occlusions of surfaces depending on their position in depth and on viewpoint. One way for the human visual system to infer monocular depth cues could be to extract and interpret occlusions. It has been suggested that the perception of contour junctions, in particular T-junctions, may be used as cue for occlusion of opaque surfaces. Furthermore, X-junctions could be used to signal occlusion of transparent surfaces.

**Methodology/Principal Findings:**

In this contribution, we propose a neural model that suggests how surface-related cues for occlusion can be extracted from a 2D luminance image. The approach is based on feedforward and feedback mechanisms found in visual cortical areas V1 and V2. In a first step, contours are completed over time by generating groupings of like-oriented contrasts. Few iterations of feedforward and feedback processing lead to a stable representation of completed contours and at the same time to a suppression of image noise. In a second step, contour junctions are localized and read out from the distributed representation of boundary groupings. Moreover, surface-related junctions are made explicit such that they are evaluated to interact as to generate surface-segmentations in static images. In addition, we compare our extracted junction signals with a standard computer vision approach for junction detection to demonstrate that our approach outperforms simple feedforward computation-based approaches.

**Conclusions/Significance:**

A model is proposed that uses feedforward and feedback mechanisms to combine contextually relevant features in order to generate consistent boundary groupings of surfaces. Perceptually important junction configurations are robustly extracted from neural representations to signal cues for occlusion and transparency. Unlike previous proposals which treat localized junction configurations as 2D image features, we link them to mechanisms of apparent surface segregation. As a consequence, we demonstrate how junctions can change their perceptual representation depending on the scene context and the spatial configuration of boundary fragments.

## Introduction

**Figure 1:**
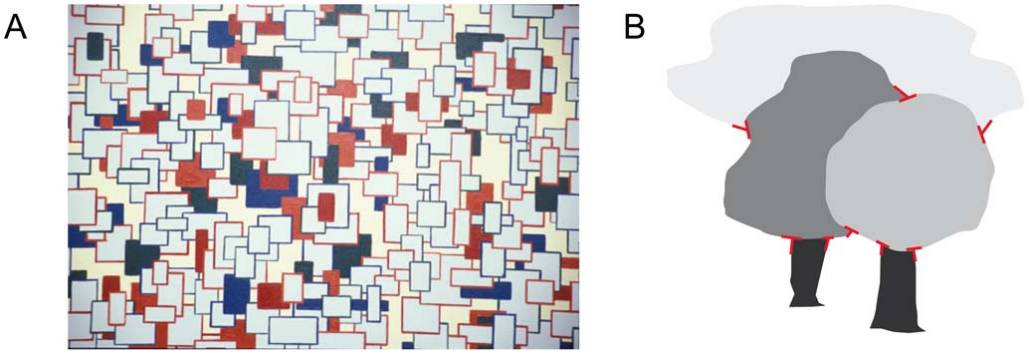
(A) Painting of a professional artist [Marrara, M., 2002, reproduction with permission from the artist] that leads to the perception of different depths induced by occlusion and colour cues. Notice how hidden surface parts are perceptually completed by the human visual system in order to segregate surfaces apart from each other. Surfaces can also be associated with (parts of) objects in scenes depicted by trees and clouds in (B). A human observer could use local cues such as T-junctions (red) formed by the boundary contour of surface parts to detect surface occlusions and hence to infer depth from monocular scenes.

Our visual system structures the visual world into surfaces that, if required, we recognize as familiar objects. A fundamental task of vision therefore is to find the boundary contours separating the regions corresponding to surfaces or objects. As our retina captures only a 2D projection of the 3D world, mutual occlusions are a natural consequence which can be interpreted by the visual system as a cue to relative depth. A vivid demonstration of surface-based depth perception is given by a painting of a professional artist who tries to depict a scene where the visual system generates surface segmentations in the presence of multiple occlusions (Figure 1). However, it remains unclear what particular features are used by the visual system to detect occlusions and whether this information is derived locally or from more global criteria. Some recent evidence [1], [2], [3] suggests that the human visual system might use surface-related features that are specific contour junctions that have a surface-based relevance in scene interpretation. In this contribution, we propose a neural model that suggests how surface-related features can be extracted from a 2D luminance image. The approach is based on contour grouping mechanisms found in visual cortical areas V1 and V2. Our computational model comprises the extraction of oriented contrasts which are subsequently integrated by short- and long-range grouping mechanisms to generate disambiguated and stabilized boundary representations. We argue that the mutual interactions realized by lateral interactions and recurrent feedback between the cortical areas considered stabilize the representation of fragments of outlines and group them together. Moreover, we demonstrate that the model is able to signal and complete illusory contours over a few time-steps. Illusory contours are a form of visual illusion where contours are perceived without a luminance or color change across the contour. Such illusory contours can be induced by partially occluded surfaces where the contour of the occluded object is perceptually completed (amodal completion) or where the occluding object has the same luminance than parts of the occluded background (modal completion). Illusory contours play a significant role in the perceptual interpretation of junction features. For instance, it was suggested by Rubin [1] that the perception of occlusion-based junctions (T-junctions) can be induced by L-junctions in combination with the presence of illusory contours. Consistently, in our model junction signals are read out from completed boundary groupings which are interpreted as intermediate-level representations that allow for the correct perceptual interpretation of junctions, namely L-junctions features can be perceptually interpreted as T-junctions. This is unlike previous approaches which are based on purely feature-based junction detection schemes [4], [5]. Taken together, our proposed model suggests how surface-based features could be extracted and perceptually interpreted by the visual system. At the same time, this leads to improved robustness and clearness of surface-based feature representations and hence to an improved performance of extracted junction signals compared to standard computer vision corner detection schemes. Based on these perceptual representations, surface-related junctions are made explicit such that they could be interpreted to interact as to generate surface-segmentations in static or temporally varying images.

## Methods

**Figure 2:**
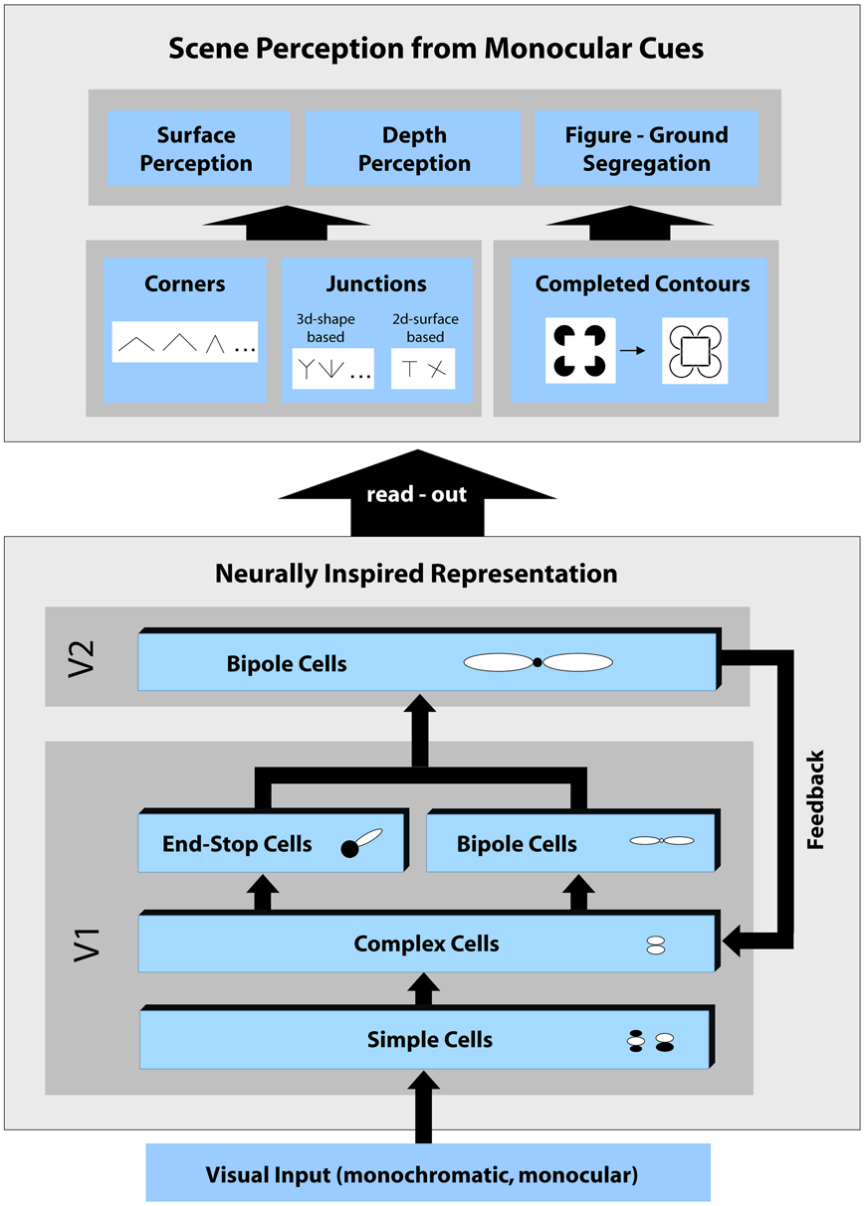
Overview of the core model architecture. The model consists of several stages that were designed to resemble properties of cells found in the early primate visual cortex. Visual input is processed by the hierarchy of different stages from visual area V1 to V2 and vice versa, that is feedforward and feedback. To enhance and complete initial contour signals, recurrent interactions between those two areas are performed iteratively until activities at all stages converge to a stable state. The converged activities can then be read-out from distributed representations to obtain specific maps that signal perceptually important image structures such as completed contours and different types of junction configurations. Such mid-level features provide important cues for occlusion detection or detection of transparencies. In addition, these mid-level features can also play a role in tasks such as border-ownership assignment which perhaps take place in higher visual areas such as V4 or IT.

In this section we give a short overview of the proposed model and its components. Our model focuses on the early processing stages of form processing in primate visual cortex, namely cortical areas V1 and V2, and incorporates hierarchical feedforward processing as well as top-down feedback connections to consider the signal flow along the reverse hierarchy processing [6]. An overview of the model architecture is depicted in Figure 2.

### Overview of the model architecture

#### The model has been structured into three main components

The first component comprises initial feedforward processing. The monochromatic input image is processed by a cascade of different pools of model cells in V1, specifically simple, complex, end-stop, and bipole cells. Each cell population consists of cells that are tuned to different orientation selectivity. This is consistent with the representation of orientation selective cells found in V1 which are arranged in hypercolumns [7]. Our model cell types are responsive for specific local image structures, i.e., simple and complex cells are sensitive to oriented contrast represented by edges or bar elements, end-stop cells respond best to contour terminations that occur, e.g., at line ends or corners, and bipole cells are sensitive for collinear arrangements of contour fragments with similar orientation. Model area V2 receives forward projections from V1 bipole cells and V1 end-stop cells. These signals are then integrated by long-range V2 bipole cells which have a larger extent than bipole cells in model V1. Bipole cells in V2 respond to luminance contrasts as well as to illusory contours, thus resembling functional properties of contour neurons in V2 [8], [9], [10]. This finding suggests that orientation selective mechanisms for contour integration in area V2 do not simply represent a scaled version of V1 mechanisms for lateral contrast integration. Their capability to integrate activities to bridge gaps and generate illusory contours makes an important step towards surface boundary segregation while V1 contrast integration is selective to stimulus feature processing. The second model component comprises recurrent feedback processing between model areas V1 and V2. Neurons in V1 are also responsive to more global arrangements of the scene [11]. These response properties possibly arise from recurrent processing and lateral connections from pyramidal neurons [12]. Whereas feedforward connections have mainly driving character, feedback connections are predominantly modulatory in their effects [13]. There is evidence that feedback originating in higher level visual areas such as V2, V4, IT or MT, from cells with bigger receptive fields and more complex response properties can manipulate and shape V1 responses, accounting for contextual or extra-classical receptive field effects [14], [15], [16]. We account for these findings by incorporating a recurrent interaction mechanism between model areas V1 and V2. In our model, activity in V2 serves as top-down feedback signal to iteratively improve initial feedforward activity in V1. The feedback signals that are delivered by descending cortical pathways multiplicatively enhance initial activities at earlier processing stages. Importantly, this type of feedback is not capable of generating new activity at positions with zero initial activity which could lead to an uncontrolled behavior of the overall system's functionality. Feedback can only *modulate* activity that is already present at V1 [12]. We shall demonstrate in our results that multiple iterations of feedforward-feedback processing between model areas V1 and V2 lead to clearly more consistent and stable results compared to purely feedforward processing schemes. The third component of the model comprises the extraction or “read-out” of scene relevant information that is provided by different pools of cells within the two model areas V1 and V2. Figure 2 presents an overview of the different types of mid-level features that can be extracted from the distributed representation of cell responses. This includes the extraction of several maps that signal contours, illusory contours, and keypoints characterized by different junction configurations. It has been stressed by several authors that specific junction configurations like T- or X-junctions provide important cues for the discovery of occlusions or transparency [1] in the context of surface segmentation. Therefore, we suggest that the visual system uses specialized mechanisms to read out separate maps for such configurations. It is important to note that our model is *not* only a simple “keypoint detector”. In fact, our model additionally provides structural information about keypoints represented by activities of model cell pools located at the keypoint.

### Detailed description of model components

**Figure 3:**
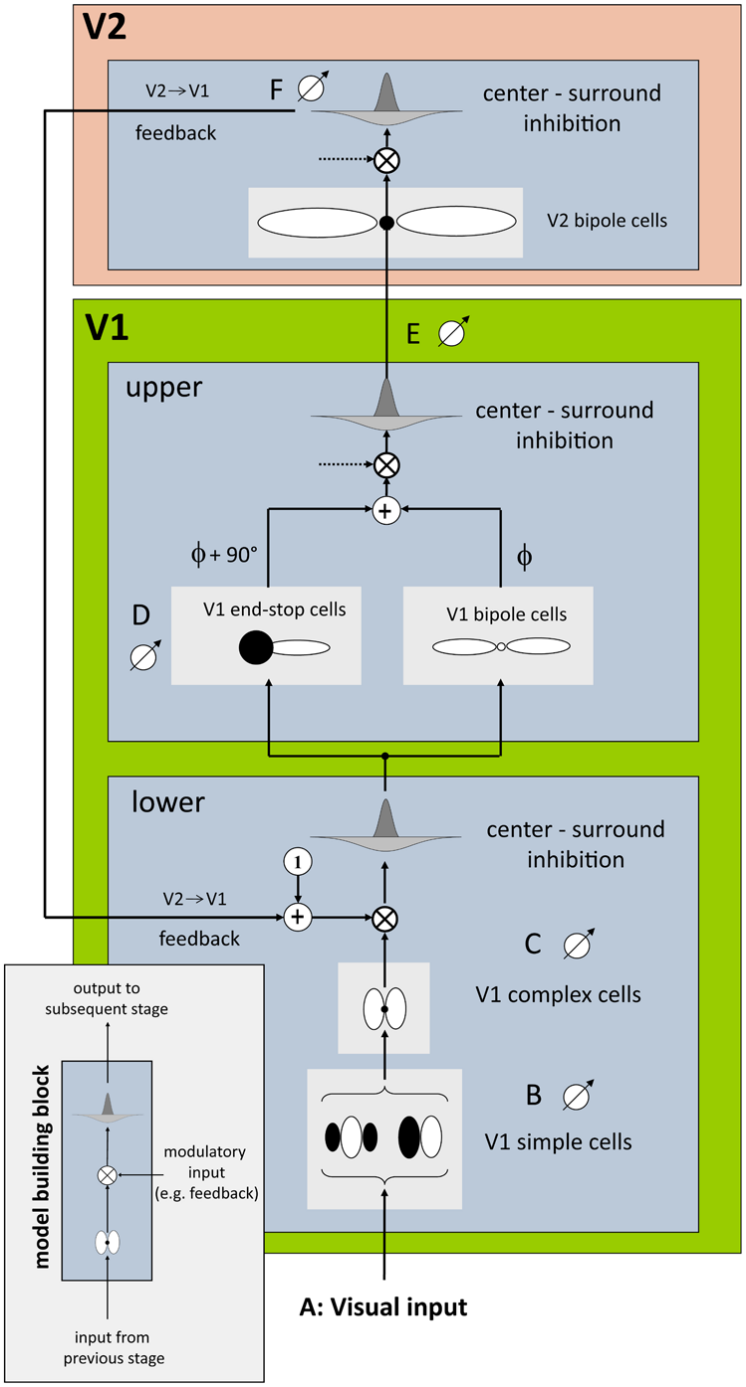
Our model simulates cells of two areas in the visual cortex, visual areas V1 and V2. Each model (sub-) area is designed with respect to a basic building block scheme (bottom, left). The scheme consists of three subsequent steps, namely filtering, modulation and centre-surround inhibition. This scheme is applied three times in our model architecture (left), corresponding to upper and lower area V1 and area V2. In this model, modulatory input (provided by feedback from area V2) is only used in lower area V1. Otherwise the default modulatory input is set to 1 (which leaves the signal unchanged). The lower part of area V1 is modelled by simple and complex cells for initial contrast extraction. Note, that each cell pool consists of 12 oriented filters equally distributed between 0° and 180°. The upper part of V1 is modelled by end-stop and bipole cells which both receive input from lower V1. The additively combined signals are further passed to area V2 where long-range lateral connections are modelled by V2 bipole cells. Note, that “•” stands for a multiplicative connection of filter subfields as employed in V2 whereas “○” stands for an additive connection as employed in V1. Finally output of area V2 is used as feedback signal which closes the recurrent loop between areas V1 and V2.

In this section, we explain the individual model parts in more detail. For a precise mathematical description of the model and its different processing stages the reader is referred to Appendix S1. The detailed model architecture is illustrated in Figure 3.

#### Model area V1

The initial feedforward stage represents early visual mechanisms in area V1 and V2 of the primate visual cortex. We do not simulate processing at earlier stages of the visual pathway such as LGN or the retina since this would not considerably influence our results. In our model, a first step is to simulate pools of cells that encode at each position of the input image oriented luminance contrasts which are represented by *V1 simple cells* in the primary visual cortex [7]. Each model cell represents the average responses (or firing-rate) of groups of neurons with similar response properties. We simulate two different types of simple cells with even and odd symmetrical receptive field (RF) properties. These orientation selective cells respond best to oriented line segments or edges, respectively. Simple cells are also selective to contrast polarity, such that they signal light and dark bars as well as light-dark and dark-light transitions. We do not, however, keep this information separate but combine this information to yield a representation of the local contrast energy, which is invariant against the sign of contrast. This is motivated by the fact that the model tries to explain computational stages to form unsigned boundaries, and thus should be invariant to contrast polarity [17]. In addition, such a convergence of activity coheres with current models of hierarchical feedforward processing of binocular input of disparity sensitive cells in primary visual cortex [18]. V1 *complex cells* pool activity of two equally oriented V1 simple cells of opposite polarity. Thus, complex cell activity is invariant to contrast polarity which resembles response properties of real complex cells. The output signals of model complex cells subsequently undergo a centre-surround inhibition that is realized by a lateral divisive inhibition mechanism. In the literature, this divisive type of lateral inhibition is also termed *shunting inhibition* [19]. This mechanism leads to a competition of cell activities within a neighborhood that is defined over the spatial and orientation domain. High activity of multiple orientations leads to a suppression of overall cell activity whereas activity in a single orientation channel leaves cell activity relatively unchanged. As a consequence, responses in areas with undirected structure such as textured or noisy areas are weakened by this operation. On the other hand, responses in areas with directed structures, such as edges and lines are strengthened by this operation. Such a stage of divisive inhibition has been previously proposed to account for non-linear effects in contrast and motion responses of V1 cells [20], [21], [22]. In the next step of the hierarchical processing scheme two different populations of cells receive forward projections from V1 complex cells. The first population of model cells resembles long-range lateral connections found in V1 [23], [24]. These long-range connections are modeled by *V1 bipole cells* which consist of two additively connected elongated Gaussian subfields. The spatial layout of the filter is similar to the bipole filter as first proposed by [17]. The spatial weighting function is narrowly tuned to the preferred orientation, reflecting the highly significant anisotropies of long-range fibers in visual cortex [25]. Here, we parameterize the length of a V1 bipole cell about 2 times the size of the RF of a complex cell. The second population receiving input from complex cells are *V1 end-stop cells*. End-stop cells respond to edges or lines that terminate within their RF. This includes also corners or junctions where more than one contour ends at the same place. However, at positions along contours or at X-junction configurations, end-stop cells do not respond. Such types of cells have been first observed in cat striate cortex [26]. More recently, evidence for end-stop cell properties of V1 neurons was found in several physiological studies [27], [28], [29]. In our model, end-stop cells are modeled by an elongated excitatory subfield and an inhibitory isotropic counterpart [30]. Our model end-stop cells are direction sensitive and are therefore modeled for a set of directions between 0 and 360 degrees. Activities of end-stop cells corresponding to opposite directions are additively combined in order to achieve direction invariance. Finally, at the output of model area V1 activities from V1 bipole and V1 end-stop cells are merged and normalized by a centre-surround inhibition stage before they are forwarded to model area V2.

#### Model area V2

Visual area V2 is the next stage in the hierarchy of processing stages along the ventral stream. Several physiological studies on macaque monkeys have shown that cells in V2 respond to luminance contrasts as well as to illusory contours [8], [31]. In contrast to complex cells in V1 they respond much stronger if the luminance contrast is continuous, and less if gaps are between inducers, thus resembling functional properties of contour neurons in V2. Moreover, they respond to moderately complex patterns such as angle stimuli [32]. However, the precise functional role of area V2 remains unclear. In our model, we employ V2 bipole cells with elongated sub-fields which are collinearly arranged and centered at the reference position. The sub-fields sample the input activations generated by V1 bipole cells and V1 end-stop cells. Responses of the individual sub-fields are multiplicatively combined [33] such that the net effect of the contrast feature integration leads to an AND-gate of the individual sub-field activations. Thus, activity from both sub-fields of an integration cell is necessary to generate cell activity. This non-linear connection has the effect that activity can emerge between two or more like-oriented contour-fragments or line ends at positions where no initial luminance contrast is present which is indicative for the presence of an illusory contour. At the same time, activity of a V2 bipole cell at an isolated contour termination would be zero as one subfield does not receive any input. V2 bipole cells are additively combined with perpendicular oriented V1 end-stop cells. This has the effect that V2 bipole cells can integrate activity of end-stop cells along line terminations that are linearly arranged, which leads to the impression of an illusory contour. Such a mechanism of *ortho-grouping* has been proposed earlier by Heitger and colleagues [8].

#### Recurrent V1-V2 feedforward-feedback interaction

In our model, lower area V1 and area V2 interact in two directions, that is feedforward and feedback. Feedforward interaction is realized by feeding bottom-up input activation from model V1 to V2 and was described in detail in the last sub-section. On the other hand, feedback interaction is realized by top-down *modulatory* feedback connections that deliver signals from model V2 to V1. The recurrent loop is closed at the feedback re-entry point in V1 where initial feedforward complex cell responses and V2 bipole cell responses are multiplicatively combined (Figure 3). In order to allow feeding input signals to be propagated, even in the case that no feedback signals exist, the feedback signal is biased by a constant unit value. This bias introduces an asymmetry for the roles of forward signals and feedback processing. Feedforward signals act as drivers of the hierarchical processing scheme, whereas feedback signals generate an enhancing gain factor which cannot on its own generate any activity at positions where no initial feeding input responses are present. This realizes a variant of the no-strong loop hypothesis [34] to avoid uncontrolled behavior of the overall model dynamics and to limit the amount of inhibition necessary to achieve a stable network performance. Several physiological studies support the view that, e.g., feedback from higher visual areas is not capable of driving cells in lower areas, but modulates their activity [12], [15]. It is important to mention that modulatory feedback in a recurrent loop only works correctly in combination with a suitable inhibition mechanism. Otherwise, the feedback signal would lead to uncontrolled growth of model cell activities. Thus, both mechanisms, the modulatory V2 → V1 feedback interaction and the subsequent shunting lateral inhibition work in combination in order to enhance distributed contour and junction representations in model V1 and V2 which mutually support each other considering a larger spatial context. At the same time, mainly through the action of the divisive inhibition mechanism, the overall activity in a pool of cells is kept within a maximum bound which stabilizes the network behavior and prevents the energy from getting too excited. In addition, those feeding activities that receive no amplification via feedback signals will be less energetic in the subsequent competition stage. Consequently, their activities will be finally reduced, which realizes the function of biased competition which has been proposed in the context of modulation in attention effects [35].

### Read-out and interpretation of model activities

**Figure 4:**
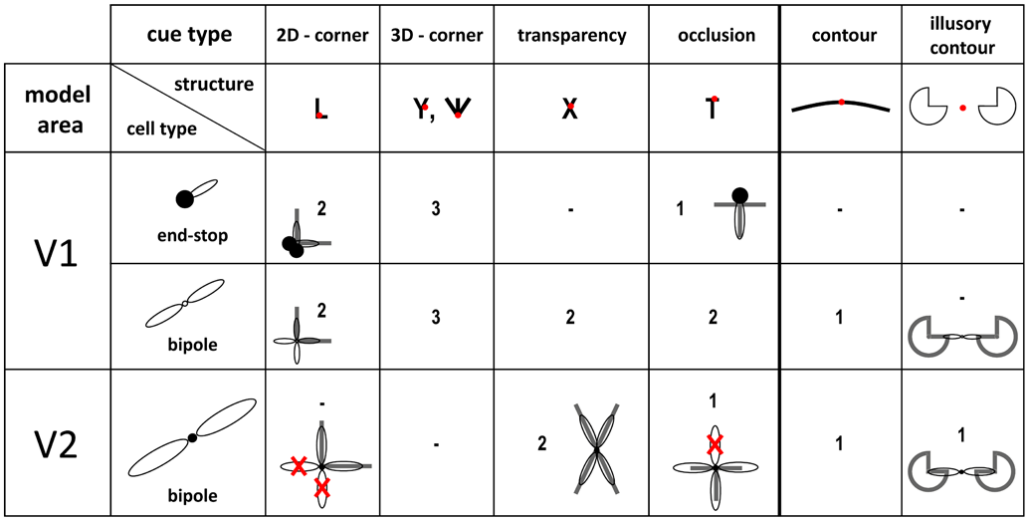
Response properties of different model cell populations for different structural configurations together with their most likely interpretation (cue type). Numbers denote the modality of the response distribution across cell pools located at the position marked with a red dot for each structure. A bar means that the cell population is not responsive for this structure. Note, that each structure has a specific neural response profile across different model cell populations which can be used to extract separate saliency maps. For a better understanding, we sketched the configuration of filters together with the underlying structure. Remember, that V2 bipole sub-fields are connected multiplicatively (signalled by a “•”), leading to zero activity of the whole bipole cell if input from one sub-field is missing (symbolized by red crosses). On the other hand, V1 bipole sub-fields are additively connected (signalled by a “○”) which has the effect that input from one sub-field is sufficient to create activity.

From the distributed representation of cell responses in both model areas V1 and V2 several retinotopic maps can be extracted that signal perceptually relevant contour configurations. If not mentioned otherwise, these maps are extracted by computing at each position the mean activity of all orientation responses. An alternative method for reading out salience values was suggested by Li [36], who choose to extract at each position the maximum activity over all orientations. In the following, we describe in detail how saliency maps for specific image structures, namely corners and junctions can be extracted by combining activities from different model cells pools. In this paper, we define saliency maps as 2d maps that encode at each position the likelihood that a specific structure is present. A more broad discussion on the concept of salience and salience maps can be found in [37]. In Figure 4 the structural configurations are sketched to present an overview of the output as signaled by the different orientation sensitive mechanisms of the proposed model. This summary indicates how the different visual structures of surface shape outlines and their ordinal depth structure might be selectively encoded neurally through the concert of responses generated by different (model) cell types. The conclusions are two-fold. First, it is indicated that the presence of, e.g., a T-junction (which most often coheres with an opaque surface occlusion [1]) is uniquely indicated by the response pattern of V1 and V2 cells at one spatial location. The T-junction is represented by an end-stop cell response at the end of the T-stem, V1 bipole cell responses in the orientations of both the T-stem (signaled by one active sub-field) and the roof, and finally a V2 bipole cell response in the orientation of the roof of the T (representing the occluding boundary). Second, we argue in favor that no explicit *detectors* are needed to represent those local 2D structures. Figure 4 indicates that the explicit representation of different junction types necessitates a rich catalogue of cells with rather specific wiring patterns. Below we propose specific read-out mechanisms in order to visualize the information we suggest is important for surface-related analysis of the input structure.

#### Contours/Illusory contours

**Figure 5:**
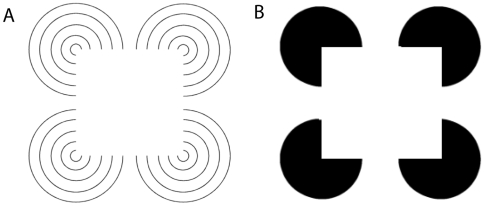
Stimuli used in our experiments that show illusory contours effects. In both, the Kanizsa shape and the five-line Varin shape, observers have the impression of seeing a white square which is partly formed by illusory contours. There is strong evidence that illusory contours are induced by horizontally and vertically arrange line-endings (A) or by collinearly arranged luminance contrasts (B).

Contours are basic image structures which are important for the segmentation of surfaces by generating a likelihood representation of the locations and orientations of the shape outline boundaries. Furthermore, contours mark the border between two adjacent surfaces and play a major role in the process of figure-ground segregation and border-ownership [37], [38], [39]. In our model, contour saliency is encoded in the response of V1 bipole cells. The contour saliency map can be extracted by summing activity of orientation selective bipole cells pools in V1. Illusory contours are a form of visual illusion where contours are perceived without a luminance change across the contour. Classical examples are the Kanizsa figure [40] where an illusory square is induced by four flanking pac-man symbols or the Varin figure [41] where linearly arranged line-ends mark the borders of the illusory square (Figure 5). There is evidence that illusory contours are represented by V2 neurons [31]. In our model, illusory contours are signaled by activity of V2 bipole cells.

#### T-junctions

A T-junction is formed when a contour terminates at a differently oriented continuous contour. T-junctions most often provide local evidence for occlusions as they frequently occur when a surface contour is occluded by another opaque surface in front. At the point where the bounding contour of the occluded surface intersects with the bounding contour of the occluding surface a T-junctions is formed in the image which is dependent on the position of the viewpoint. It has been suggested by Rubin [1] that T-junctions play a central role in monocular depth perception, surface completion, and contour matching. To read-out T-junction signals we multiplicatively combine activities of V2 bipole cells with V1 end-stop cells. More precisely, we use a pairwise combination of orientation specific V2 bipole cell activities and orientation specific V1 end-stop cell activities. Pairs of cells that correspond to same orientations are not considered. The multiplicative operation realizes an AND-gate, implying that both cell populations have to be active in order to signal the presence of T-junctions. A saliency map that signals the presence of T-junctions is then extracted by summing over the orientation domain. In general, this map represents the priority of the gathered evidence in favor of the particular scene feature.

#### L-Junctions

**Figure 6:**
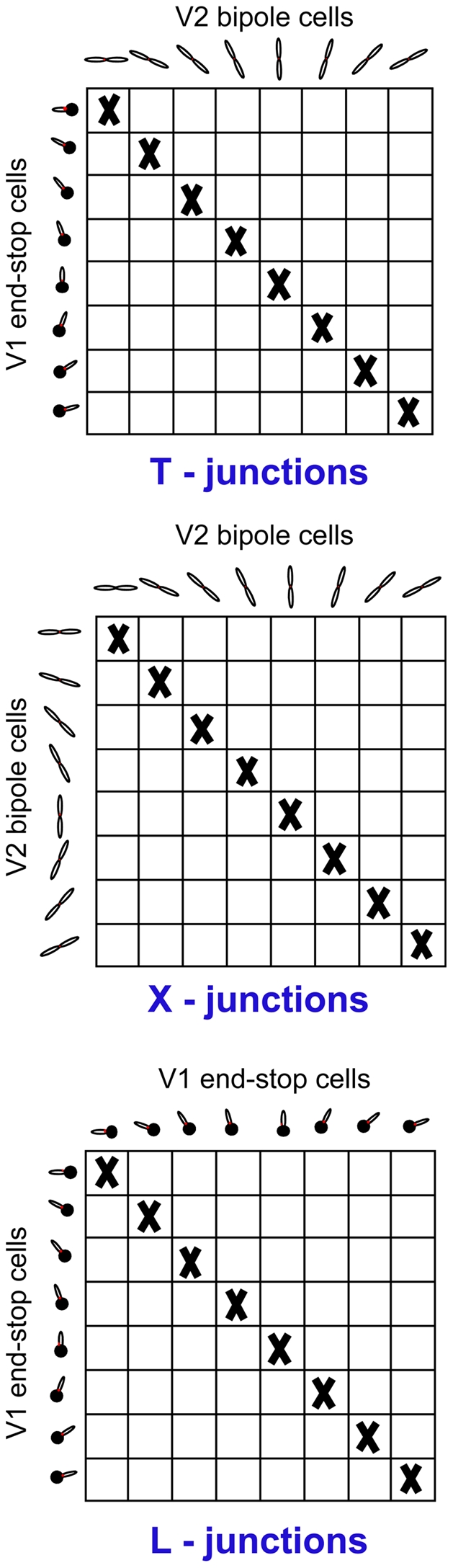
Extraction of junction signals. The figure depicts how local activity from model cells is combined to obtain specific junction maps. To extract a T-junction signal orientation specific V2 bipole cell activities are combined pairwisely with orientation specific V1 end-stop cell activities. Pairs of cells that correspond to same orientations are not considered (diagonal marked with “X”). The combination of cell activities is done multiplicatively such that both cells have to be active in order to produce a response. This can be represented in a map where each entry corresponds to a specific T-junction configuration. X-, and L-junction maps are extracted similarly except that pairwise combination is based on V2 bipole cells (X-junctions) and V1 end-stop cells (L junctions). To summarize, the maps represent information about local image structure with respect to different junction types. Note, that end-stop cells are only indicated for one direction in the figure. However, end-stop cell responses of both directions that correspond to one orientation are additively combined for the extraction of junction maps.

L-junctions, also termed V-junctions or corners, are formed by two contour segments which terminate in the same projected location. We extract corner signals in a similar way than T-junctions signals. Instead of combining V1 end-stop activities with V2 bipole activities we multiplicatively combine activities of differently oriented V1 end-stop cells among each other (Figure 6). A combined orientation invariant corner saliency signal is then extracted by integrating over all combinations. Corners are important cues for shape perception as they mark keypoints of the boundary contour. Importantly, an L-junction can also result from two occluding surfaces, assuming that one of the surfaces is partly formed by illusory contours [1]. Under such circumstances, an L-junction feature would as well suggest for occlusions. We shall show that our model initially detects an L-junction in this case, but over time when groupings could be established, then these types turn into perceptual T-junctions (irrespectively whether the boundaries are formed by physical luminance contrasts or by illusory contours). The perceptual representation of T-junctions in turn signals the presence of an occluding surface which is consistent with the impression that human observers report when they are confronted with illusory figures, such as Kanizsa squares [40].

#### X-junctions

X-junctions configurations appear at positions where two contours intersect. In scenes with overlapping surfaces, this pattern is created when an occluding surface has transparent material properties leading to a visibility of the occluded surface region through the surface at the occluded surface contour. Therefore, in the transparent occlusion situation, a T-junction turns into an X-junction. In our model, X-junctions are read-out by multiplicatively combining activities of pairs of differently oriented V2 bipole cells (Figure 6). The saliency map is obtained by summing over the orientation domain. Again, the multiplicative connection acts like an AND-gate which extracts only those V2 bipole responses that have a bimodal activity distribution in the orientation domain.

#### Y- and W-junctions

Y- and W-junctions are strong cues for 3D-corners induced by surface intersections of 3D objects which cut in a single location. For instance, a cube produces a Y-junction at the position where the corners of three visible surfaces meet. The same corner observed from another viewpoint turns into a W-junction. Notably, in rare cases, such junctions can be also produced by occluding 2D surfaces when a contour of an occluded surface meets a shape-based L-junction of an occluding surface. However, this can be seen as an ‘accidental’ and rather unstable configuration since even small changes in viewpoint would lead to vanishing of such occlusion-based junctions. Since the perception of 3D objects is not our primary focus in this contribution, we do not take Y- and W-junctions into further consideration.

#### Competition between junction signals

**Figure 7:**
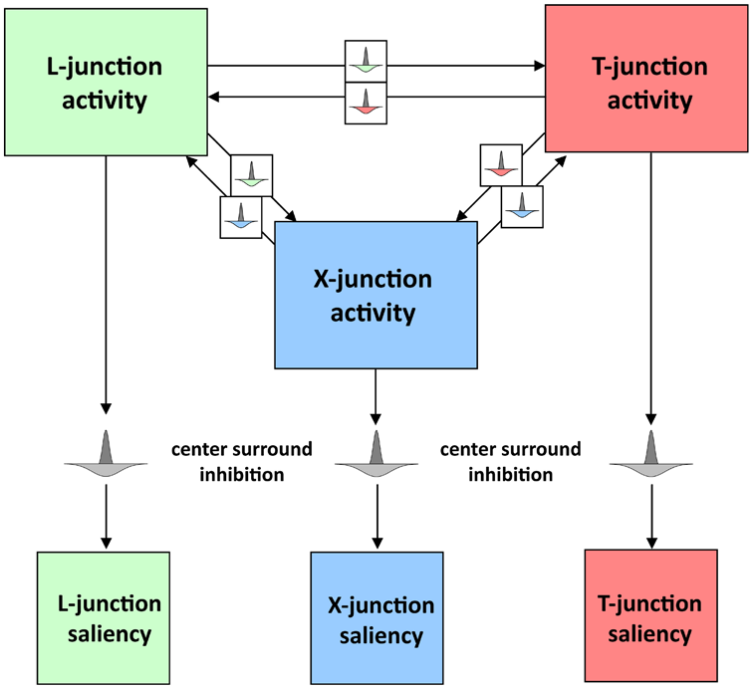
Competition between junction signals. Junction signals can locally compete with each other to avoid ambiguous signals. In addition, centre-surround inhibition helps to suppress multiple junctions in a small neighbourhood which could be induced by fine texture or noise.

In order to suppress ambiguous activations for more than one junction type at the same place, junction signals compete with each other through lateral inhibition (Figure 7). If one junction type is activated the other junction signals in a local neighborhood are weakened. Finally, all junction activities are passed through a non-linear saturation function in order to have the same range for all activity signals. Note, that although we use a similar inhibition scheme than for the model activities we do not claim that this kind of competition has a biological counterpart. This is just a necessary operation to disambiguate feature signals and has no biological relevance.

## Results

In this section we present results of the model simulations in order to demonstrate the computational capabilities of the proposed model. We begin by presenting model results from the various neural cell pools that are simulated in the model. We also show how recurrent feedforward-feedback interaction helps to enhance and stabilize the initial responses of cells. Then we show that various feature maps can be extracted from the distributed cell activities suggesting that this representation is capable of providing cues that are perceptually relevant for fundamental visual processes such as occlusion detection.

### Robustness to noise

**Figure 8:**
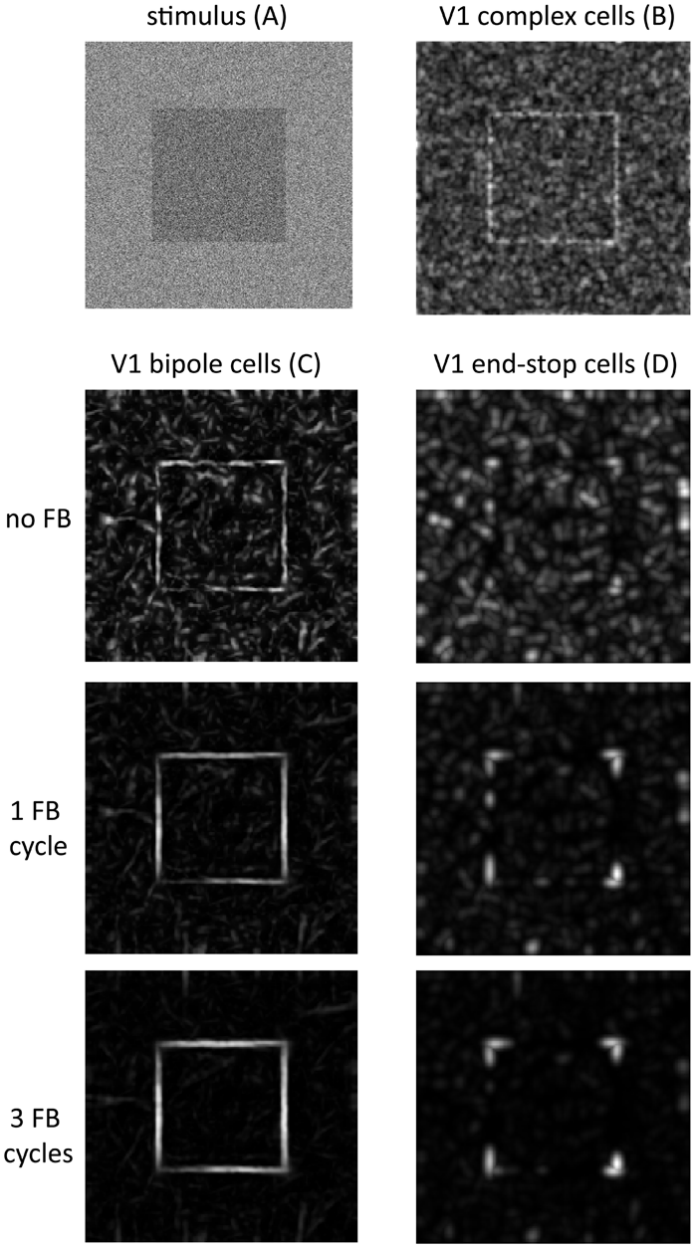
Robustness to noise. A square stimulus that has been corrupted with high amplitude Gaussian noise was used as model input (A). Initial complex cells responses are strongly influenced by the noise pattern (B). Recurrent feedforward-feedback processing significantly reduce activity of noise-induced responses of V1 bipole cells (C) and end-stop cells (D) (illustrated are responses after 0, 1, and 3 cycles of feedback). At the same time, surface contours and corners are enhanced over time.

In a first simulation an artificially created image of a noisy square is employed to demonstrate the robustness of the model against noise perturbations. The image was created such that the standard deviation of the additive Gaussian noise equals the luminance difference of the square against the background (so-called 100% noise). Figure 8 shows that initial complex cell responses are strongly disrupted resulting from the additive noise pattern. However, recurrent feedforward-feedback iterations lead to a significant reduction of noise responses and at the same time to a strengthening of contour and contour-termination signals corresponding to V1 bipole and V1 end-stop cell activities, respectively. Note that the long-range interaction stage does not lead to activities beyond contour terminations at the corners of the square which would mistakenly lead to turn L-junctions into X-junction.

### Extraction of junction configurations

**Figure 9:**
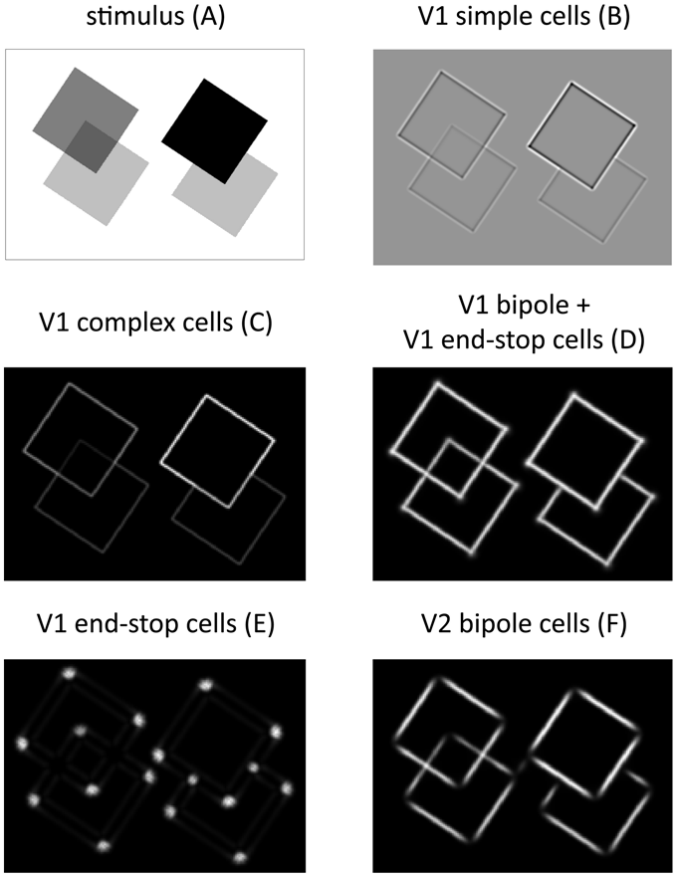
Model responses based on artificial input stimulus of overlapping squares. The stimulus (A) includes two different occlusion conditions: two overlapping opaque surfaces that produce T-junctions and two overlapping transparent surfaces which generate X-junctions. Furthermore, several L-junctions are visible at the corners of the squares. Model activities are summed over the orientation domain for all stages of our model. We show initial V1 simple and complex cell responses (B, C), combined V1 bipole/end-stop cell responses (D), end-stop activity (E), and V2 bipole cell activity (F) after 4 recurrent feedforward-feedback cycles. For clarification, the labels (A)–(F) correspond also to labels (A) – (F) in Figure 3. Note that end-stop cells do not respond to X-junction configurations and that V2 bipole cells do not respond at L-junctions (cp. Table 1). Note also, that responses in (D) – (F) are invariant against image contrast.

In a second simulation, we used a noise-free image of four occluding transparent and opaque squares as input for the model (Figure 9a). The stimulus includes all three types of junction configurations and is therefore a good example to demonstrate the capabilities of the model at a glance.

Initial feedforward responses from simple and complex cells shown in Figure 9b and Figure 9c demonstrate that these responses are not invariant to luminance contrast. Furthermore, they are not robust against noise (Figure 8). The results described in the following correspond to model activities after four recurrent feedforward-feedback cycles where model activities tended to converge to a stable state. The upper sub-area of model V1 is represented by bipole cells and end-stop cells. End-stop cells respond at positions where contours meet (e.g. T-, and L-junctions) or at positions where a contour ends (Figure 4). However, they do not respond at X-junction configurations as can be observed in Figure 9e. Model V1 bipole cells are responsive for contours. They connect short like-oriented fragments and equalize contrast changes along the contour. At contour endings their activity is reduced (not shown) since they have additively connected subfield (one subfield is still activated, see Figure 4). The additive combination of V1 bipole and perpendicular oriented end-stop cells compensates for the reduction effect at corners or T-junctions. Figure 9d show that contour activity is not reduced at corners or T-junctions. The combined bipole end end-stop activities from model area V1 are further processed by V2 bipole cells which are as well responsive for contours. However, as a result of their multiplicatively connected subfield these cells do not respond at contour endings. Consequently, V2 cell responses are zero at T- and L-junctions, but not at X-junctions (Figure 9D, Figure 4).

**Figure 10:**
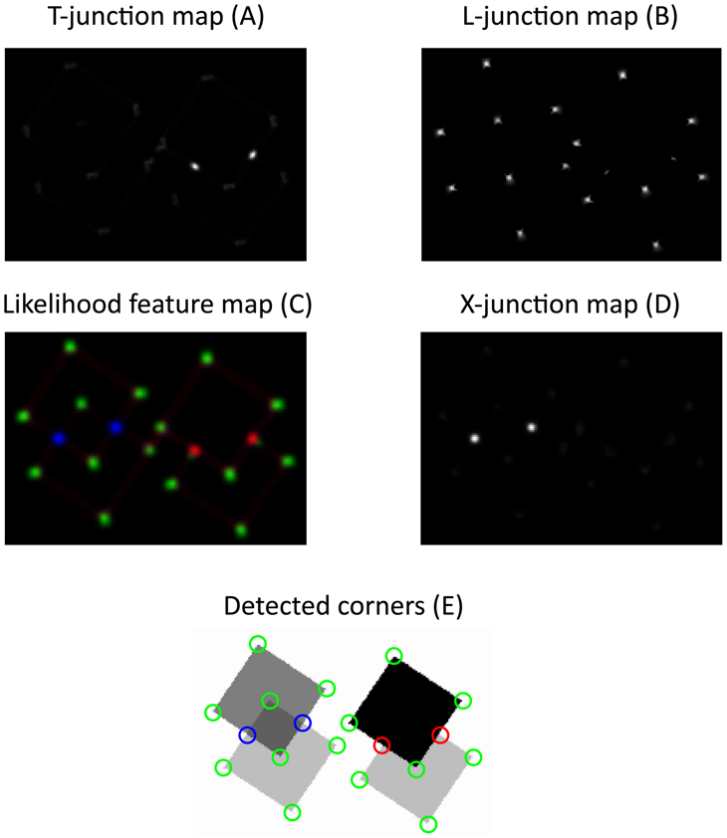
Saliency maps for different junction types. The junction maps (A), (B), and (D) were extracted from model activities presented in Figure 9. Bright regions indicate positions where the ensemble of model activities suggests for the presence of the respective junction type (L-, T-, X-junction). A combined feature likelihood map(C) incorporates all features, colour coded. Blue signals the presence of X-junctions, red signals T-junctions and green signals the presence of L-junctions. Moreover, position and type of detected features is visualized in (E) (based on maximum likelihood selection).

In Figure 10, T-,L-, and X-junction maps were extracted and visualized based on converged model activities. In each map, the presence of the specific feature is signaled by patches of high activity. In order to prevent multiple features to be active at the same position, all signals undergo a competition stage where multiple signals in a local neighborhood compete with each other. The output of this stage is presented in Figure 10c which is a combined map that represents different features, signaled by color. Finally, from this map, position and type of junctions are extracted by local maximum selection (Figure 10e).

### Processing of illusory contours

**Figure 11:**
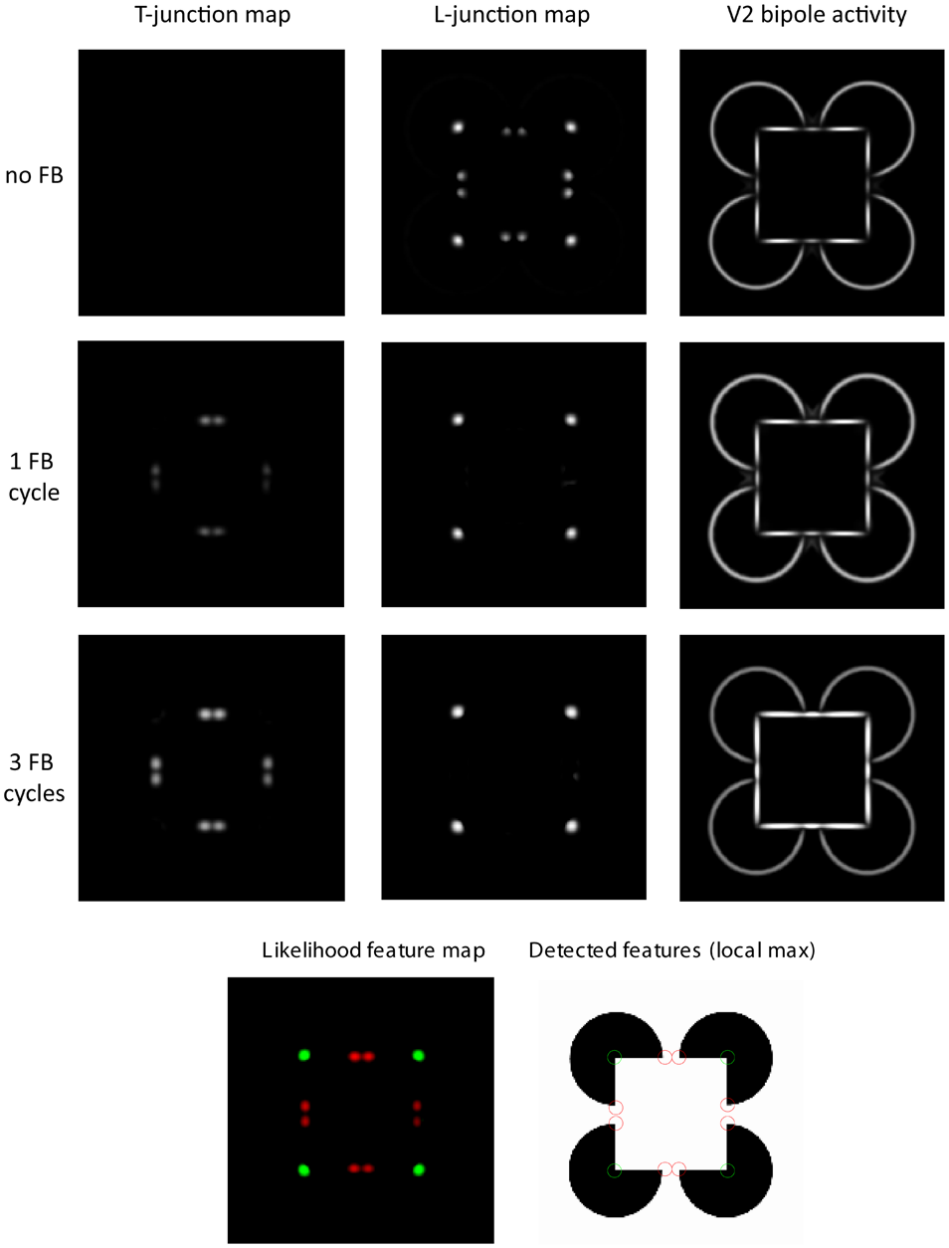
Recurrent processing of illusory contours. A Kanizsa figure is used as input for the model. V2 bipole cell activities and T-/L-junction signals are illustrated initially (no recurrent processing), after one recurrent cycle and after 3 recurrent cycles. The combined feature map show individual likelihoods colour coded. Local maxima of the feature map are used to detect junction positions. Illusory contours are signalled by V2 bipole cell activities and are completed over time. Note, that as a result of the completion process of illusory contours, L-junctions signals turn into T-junction signals over time.

**Figure 12:**
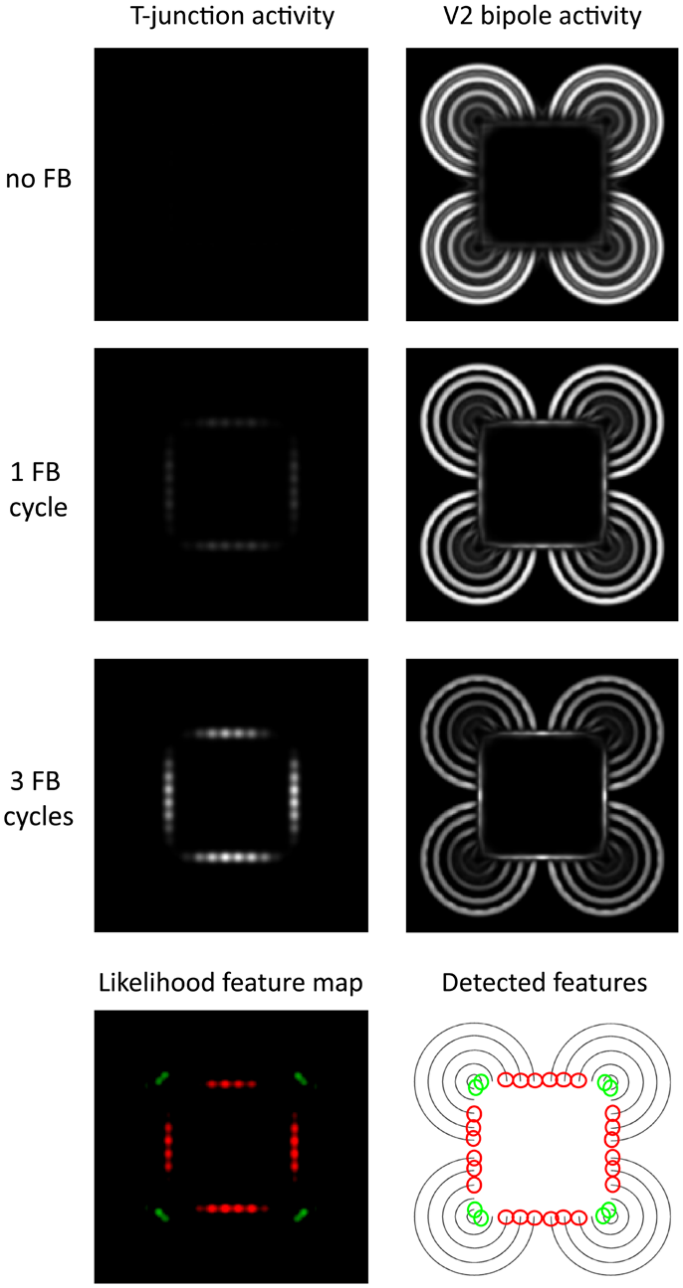
Recurrent processing of illusory contours formed by line ends. An alternative version of the Kanizsa figure is used where line ends lead to the impression of an illusory white square. Model responses are visualized according to Figure 8. Note, that illusory contours signalled by V2 bipole responses develop over time. Note also, that over time this leads to the development of T-junction signals (red) which suggest for the presence of an occluding surface.

In a third simulation, we show the capabilities of the model to uncover illusory contours in scenes. As input we used two different versions of the Kanizsa square [40](Figure 5). The first image leads to the impression of an illusory square where the corners occlude black circles. The second image gives the impression of a white square in front of concentric circles. In both cases, only parts of the square are formed by luminance contrast. However, human observers mentally *see* the square as a coherent object. Our model results demonstrate that the invisible contour parts are uncovered by V2 bipole cell responses. Moreover, we show that subsequent recurrent feedforward-feedback cycles help to close large gaps between like-oriented contour elements or along linearly arrange contour endings (Figure 11, Figure 12).

Importantly, this has a strong influence on the junction type that is signaled by the model responses. In Figure 11, initial model responses suggest for L-junctions, which is in accordance with the physical luminance contrasts. But, after some recurrent iterations, V2 bipole cells close the gaps between contour elements which leads to a different, more global interpretation: the L-junctions along the illusory contour turn into T-junctions. The emergence of T-junctions in turn supports the perceptual interpretation of occlusions.

A similar effect can be observed in Figure 12 where line endings initially produce no junction signals. After a few feedforward-feedback cycles, however, the emerging illusory contour responses of V2 bipole cells lead to T-junction signals along the contour.

### Processing of real-world data

**Figure 13:**
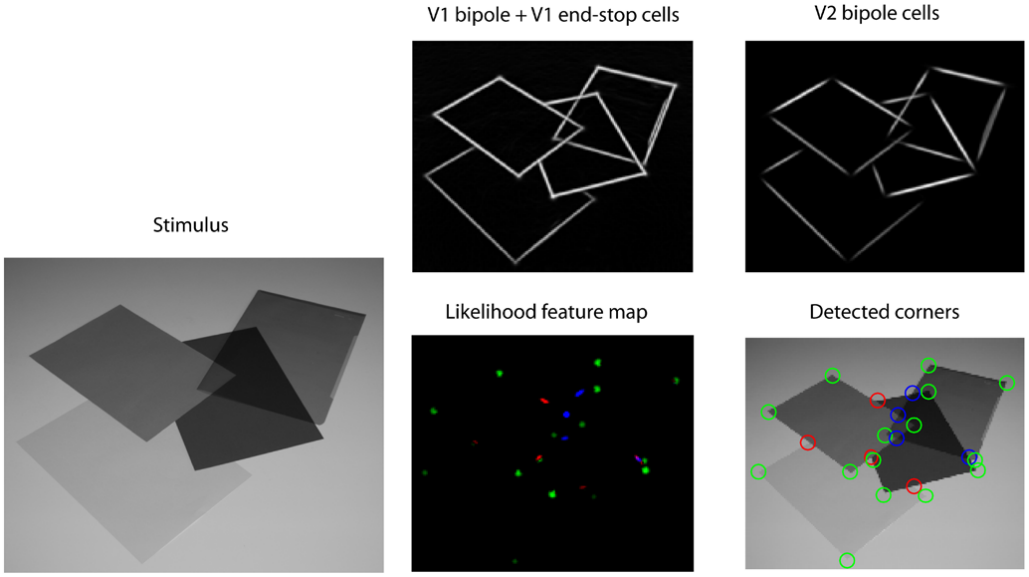
Model activities of V1 and V2 cell pool (right, first row) resulting from a real-world scene image that includes occluding opaque and transparent surfaces. A feature map was extracted from model activities, revealing position and type (colour coded) of junction configurations (right, second row).

In order to examine how the model performs for real-world camera images we used an image taken from a desk scene where several papers and a transparent foil are arranged such that they partly occlude each other (Figure 13). Model activities and the extracted feature map demonstrate that the model is also capable of dealing with real-world images. Note, that one of the papers has a very low contrast ratio with respect to the background. Nevertheless, the model performs excellent in finding the contour and the respective junctions. This also underlines that the model is invariant to contrast changes and thus also stable against changes of illumination conditions.

### Quantitative evaluation and comparison

**Figure 14:**
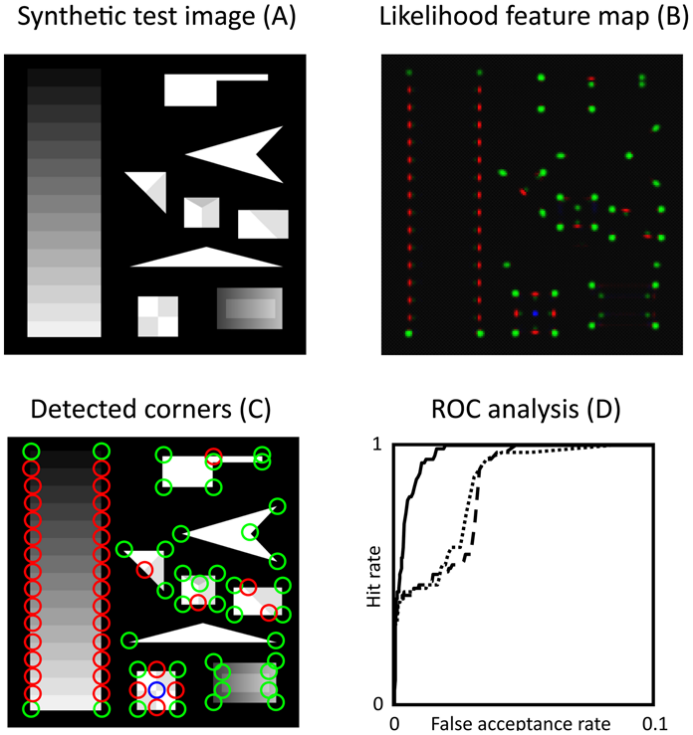
Evaluation of extracted junction signals on synthetic test image. A synthetic test image (A) reproduced from Smith and Brady (1997) was used to evaluate and compare extracted junction signals against a computational scheme (structure tensor) for corner detection proposed by Harris and Stephens (1988). The extracted junction saliency is visualized in a feature likelihood map (B) and detected junction positions/types are superimposed on input image (C). ROC curves are computed from structure tensor results (dashed), initial model responses (dotted) and from converged model responses after 4 recurrent cycles (solid) (D). Abscissa denotes the false alarm rate, and the ordinate denotes the hit rate. Note, that for better visibility the abscissa has been scaled to [0, 0.1].

In this section, we evaluate our model by comparing our recurrent junction detection scheme with results obtained by simply switching the recurrent feedback cycle off, which reduces the model to an ordinary feedforward model. Moreover, we compare our results to a standard computer vision corner detection scheme based on the Harris corner detector [4]. In a comparative study of different corner detection schemes, the Harris corner detector provides the best results among five corner detectors [42]. As input for our comparison, we use corner test image adapted from Smith and Brady [5] that poses several challenges such as, e.g., low contrast regions, smooth luminance gradients, or obtuse and acute angles. Moreover, all types of junctions (L, T and X) considered are represented in the test image together with information about their exact position (ground truth information). Since the Harris corner detector is not able to discriminate between different junction types, our comparison is only based on the detection performance, irrespective of the junction category. To measure the performance of the different schemes we use receiver operator characteristic (ROC) curves. This method is frequently used to evaluate true positive rate or hit rate and the false positive rate of a binary classifier system as its discrimination threshold is varied. Here, we use the junction feature map as input for the ROC analysis. Figure 14 shows the resulting ROC curves extracted from junction feature map given the test image as input. It is clearly visible that the ROC curve computed from the recurrent model responses lies well above the Harris corner detector curve and the initial feedforeward model curve. This suggest for a significantly better detection performance of our recurrent model compared to feedforward processing-based junction detection schemes.

### Simulations with dynamic input stimuli

We particularly designed this experiment to demonstrate a model prediction, namely, that feedback leads to a brief persistence of object and material appearance. This is usually unnoticed when the scene does not change at all. If, however, appearances of surface material change during recurrent interaction (while keeping the shape registered) the apparent surface property should stay more prolonged depending on whether it is transparent and changes into opaque or whether it is opaque and smoothly changes into transparent. Due to the action of modulatory feedback activation the registered boundary and junction activations continue to enhance the configuration signaled from previous input features for a short period of time. This appearance history (or memory in the processing architecture) is known as hysteresis effect.

Here, we used temporal sequences of two occluding squares where the opacity of the topmost square was linearly altered between 100% opacity and 90% opacity, thus making the occluded region increasingly more visible (invisible). The input sequence consists of 10 frames static input (opaque) followed by 10 frames linear change from 100% to 90% opacity followed by 10 frames static input (transparent, 90% opacity). The sequence was presented as described above (opaque-transparent) and in reverse temporal order (transparent-opaque). To investigate the hysteresis effect of feedback we presented both sequences to the full model (with feedback connection enabled) and to a restricted version of the model with feedback connections disabled. In the full model, feedback processing time is equal to stimulus presentation time, i.e., one feedback cycle is performed per stimulus time frame. In the restricted model, we switch all feedback connection off which constrains the model to perform only feedforward processing.

**Figure 15:**
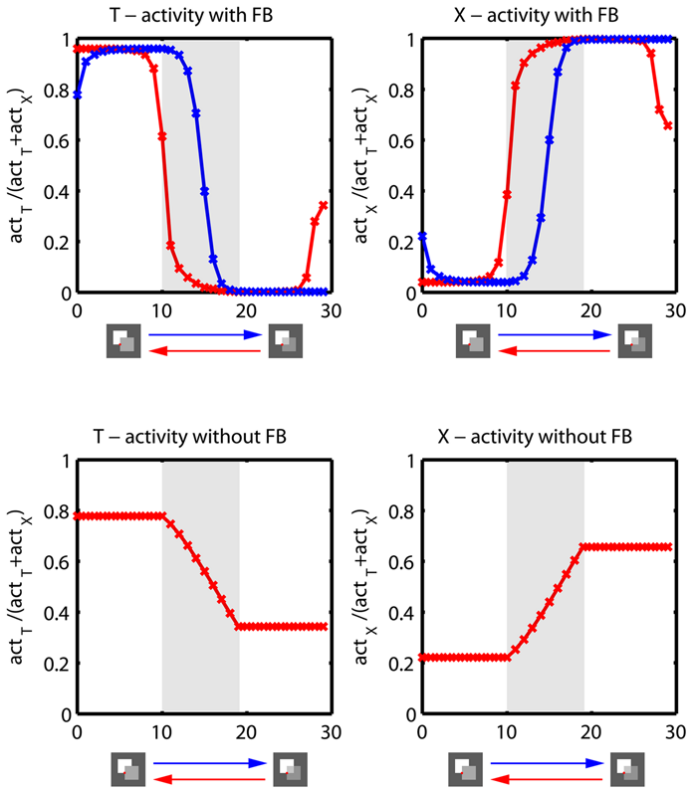
Hysteresis effect induced by feedback. The figure illustrates T- and X-junction activities extracted from the model based on temporal input sequences of two occluding squares where the topmost square changes material appearance from opaque (100% opacity) to transparent (90% opacity) (blue lines) over time. Furthermore the sequence was presented in reverse temporal order leading to a change of material appearance back from transparent to opaque (red lines). Activities were extracted at positions indicated by the red dot on the stimulus. Illustrated are results based on model simulations with feedback (top row) and without feedback connections (bottom row). Gray shaded areas indicate periods where stimulus properties linearly change. The results demonstrate that feedback leads to a hysteresis effect by temporary locking the prediction for a junction type (T- or X-junction). Without feedback the hysteresis effect disappears and both input sequences produce exactly the same results (only red curve is visible). Furthermore, the results demonstrate that feedback helps to disambiguate initial junction signals by amplifying the most likely prediction and suppressing weak predictions over the first few iterations.

Throughout the simulation, model activities indicating T- and X- junctions are extracted at positions where occluding contours of both squares intersect each other (Figure 15). The results generated by the full model show that feedback leads to a sequence directional hysteresis effect by temporally locking the prediction of a junction type (T- or X-junction). Moreover, initial ambiguities induced by predictions for different junction types are resolved by top-down feedback during the first few iterations. In contrast, when the model is restricted to feedforward processing no hysteresis effect can be observed, i.e., the model activities are equal for both input sequences, namely opaque-transparent and transparent-opaque transitions. Furthermore, no disambiguation between different junction type predictions takes place.

## Discussion

In this section we begin by summarizing our main findings. Then, we compare our model with other proposed models that are related to our work. Moreover, we show that all core mechanisms employed in our model are biological plausible. We also discuss how junction signals that are extracted from our neural representation could be used by other cortical areas to solve visual tasks such as depth ordering, figure-ground segregation or motion correspondence finding. Finally, we briefly discuss some examples where our model fails to produce accurate results.

### Summary of findings

We presented a recurrent model of V1-V2 contour processing utilizing long-range interactions in combination with short-range lateral inhibition which as been adapted from [33]. We have shown quantitatively and qualitatively that recurrent combination of contextual features substantially enhances the initial estimates of local contrast.

We have also shown that the model V2 cells are capable of generating illusory contour groupings which strongly influence the interpretation of different junction type estimates of local contrast.

In addition, we demonstrated that cell activities represented in both model areas can be combined and extracted to robustly signal three different sorts of junctions (L-,T-, and X-junctions). These junctions provide important cues for fundamental visual processes such as surface completion and figure-ground segregation [1], [39], [43]. Furthermore, our model predicts a hysteresis effect between opaque-transparent and transparent-opaque transitions which could be experimentally validated in a psychophysical experiment. Finally, we have demonstrated in a quantitative analysis that our model responses outperform a state-of-the-art computer vision corner detection scheme.

### Related work

A number of different models have been proposed for contour integration. A comprehensive review can be found in [44]. Our contour integration model which utilizes interaction of feedforward and feedback, in particular modulatory feedback, has been applied successfully to a number of different tasks of visual processing such as optical flow estimation [45], texture processing [46], selective attention [47], cortico-thalamic enhancement [48], and linking synchronization [49].

One of the first computational models in the context of contour grouping that incorporate principles of long-range interactions and *interlaminar* recurrent processing has been proposed by Grossberg and colleagues [17] by introducing the Boundary Contour System (BCS). A more recent version of the BCS focuses more on the *intercortical* processing between areas V1 and V2 [50]. Grossberg and coworkers propose that V2 is mainly a slightly modified version of V1 operating at a coarser scale. Thus, they suggest that both areas, V1 and V2 share the same functional properties. Unlike them, we argue that V1 and V2 have different functional roles, e.g., corner selective cells occur in V1, bipole cells responding to illusory contours occur in V2. A more fundamental difference in the model of Grossberg and colleagues is that they use additive feedback connections with the effect that new activity can be generated in model area V1 at positions where initial bottom-up signals are zero. To compensate for this, they have to incorporate thresholds which lead to more complex balancing processes. However, we use modulatory feedback connections, which implies that initial bottom-up activity is *required* to generate activity. Thus, in our model, illusory contours characterized by zero luminance contrast can only be signaled in V2, but not in V1. This is consistent with electrophysical studies of Peterhans and von der Heydt [51] who concluded that illusory contour cells are virtually absent in V1. Concurrently, there is strong evidence for illusory contour selective cells in V2 [9]. A recurrent model of V1-V2 interactions based on modulatory feedback was first proposed by Neumann and Sepp [33].

Mundhenk and Itti [52] presented a multi-scale model for contour integration that is motivated by mechanisms in early visual cortex (V1). Similar to our model, the authors try to extract saliency values from contour representations. Individual contour saliency maps from different scales are combined by weighted averaging. Differences to our model are that they do no incorporate feedback mechanisms and that they do not consider illusory contour extraction as they do not model V2 neurons.

Interestingly, only few computational models of contour grouping address the computation and representation of corners and junctions. The model proposed by Heitger et al. [8] is closely related to our model because they use several elements that we incorporated into our model (e.g. complex cells, end-stop and bipole operators). A key element of their model is the concept of *ortho-* and *para-*grouping to generate illusory contour representations. *Ortho* grouping applies to terminations of the background, which tend to be orthogonal to the occluding contour. *Para* grouping applies to discontinuities of the foreground and is used to interpolate the contour in the direction of termination. However, a major shortcoming of their model is that it relies on a purely feedforward scheme which would presumably produce erroneous results when given a degraded input image (e.g. by noisy or low contrast). This also contrasts with the findings of several authors [1], [12], [15] [37] [53] that feedback and recurrent interactions play an important role in visual processing for figure-ground segregation.

The model of Hansen and Neumann [54] is also closely related to our model since it is based on the same biological principles such as modulatory feedback and long-range interactions for the extraction of corners and junctions. However, the model is restricted to interlaminar interactions in V1 to explain contrast detection and subsequent enhancement effects. Our model, on the other hand, incorporates several extensions. First, our model takes illusory contours into account by additionally modeling area V2. Second, we show that our neuronal representation is further processed to extract different junction types such as L, T- and X-junctions which are perceptually important features that provide basic cues for global scene interpretations.

Recently, a recurrent model for surface-based depth processing was proposed by Thielscher and Neumann [30]. In their proposed model, depth information derived from monocular cues is propagated along surface contours using local recurrent interactions to obtain a globally consistent depth sorting of overlapping surfaces. The model differs in several aspects from our model. In contrast to our model, they use additional recurrent interactions in V2 to propagate border-ownership information derived from detected T-junctions along contours. This propagated information enables them to obtain a globally consistent interpretation of depth relations between surfaces. Unlike this approach to monocular depth segregation, we focused on the extraction and perceptual interpretation of junction configurations.

In summary, variations of mechanisms employed in our model can be found in several other models of visual processing. But only few of them have concerned for combined boundary extraction and junction extraction as well as their distinction. Unlike previous proposals which treat localized junction configurations as 2D image features [4], [5], we link them to mechanisms of apparent surface segregation. As a consequence, we demonstrate how junctions can change their perceptual representation depending on the scene context and the spatial configuration of boundary fragments.

### Biological Plausibility

Our model architecture is inspired by biological mechanisms and is based on neural representations of early visual cortex. We now put individual model components into a physiological or psychophysical context and discuss for their plausibility.

#### Biological plausibility of model components

In the initial stages of our model we simulate V1 simple and complex cells [7]. Model V1 bipole cells are inspired by horizontal long-range connections that link patches of neurons of similar orientation preference [23], [25]. Consistently, model V1 bipole cells pool activities of appropriately aligned complex cells from the lower part of model area V1 (Figure 3) which resembles intracortical layer 4 of area V1. Evidence for nonlocal integration also comes from psychophysical experiments for contrast detection [55] and contour integration [56]. Additionally, we model end-stop cells that selectively respond to contour terminations. The existence of V1 neurons reacting to end-stop configurations has been confirmed by several electrophysiological studies [26], [57], [58]. As a consequence, end-stop cells were also modeled by several authors in the context of contour integration [30], [51], [59].

In model area V2, we employ modified bipole cells with nonlinear response properties. As V2 neurons have larger receptive fields than V1 neurons, our bipole filters employed in model area V2 have a larger extent that those used in the upper part of model area V1 (Figure 3). Evidence for contour selective cells in V2 comes from von der Heydt [9] where the authors probed V2 neurons with illusory-bar stimuli. They selectively respond to coherent arrangements having both fragments of an illusory bar intact. If one fragment is missing, the cell response drops to spontaneous activity [51]. To be consistent with these findings we modeled V2 bipole cells with multiplicatively connected sub-fields which leads to similar effects than those reported by von der Heydt.

#### Evidence for representation of junctions and corners in visual cortex

Although it seems obvious that junctions play a crucial role in several perceptual processes [1] only little evidence was found that specific cells in the visual cortex are particularly responsive to junction features.

Several studies suggest the presence of a neural organization in V1 that may represent a mechanism for detecting local orientation discontinuity [60], [61], [62]. Their results indicate a facilitory interaction between elements of V1 circuitry representing markedly different orientations in contradiction to the common believe that functional connectivity is only seen between cells of like orientation [63]. However, it is still unclear to what extent this selectivity is used for junction processing.

In a study by Kobatake and Tanaka [64] critical features for the activation of cells reaching from V2, V4 up to posterior inferotemporal cortex (IT) were determined. V2 cells were found to react to stimuli such as concentric rings or tapered bars. Cells that respond selectively to junction-like features like crosses were only found in V4 and posterior IT.

More recently, two studies [32], [65] report on cells in monkey visual area V2 that seem to explicitly encode combinations of orientations as represented by junctions or corners. Thus, such V2 neurons may provide important underpinnings for the analysis of surfaces [2]. In a straightforward model approach it was shown that these V2 neurons may simply sum the responses from orientation selective V1 neurons [66]. However, the fact that only little evidence exists for junction selective cells in V2 could also motivate the hypothesis that junctions are not explicitly encoded by specific cells in V2 but higher visual areas such as V4 or IT link responses from cell types selective for lower-level features, such as complex, end-stop, and bipole cells. Thus, the extraction of junction signals from combinations of model cell responses, described as *read-out process* in our model, follows the idea mentioned above, that, e.g., V4 cells could pool signals from several cortical areas, particularly from V1 and V2. Notably, we do not claim that junction signals are encoded by V4 or IT neurons, but we demonstrate that our model performs well assuming that junctions are processed from distributed activities of neurons at early cortical stages.

#### Model predictions for psychophysical experiments

Our model incorporates recurrent feedback processing from higher to lower stages. This leads to temporal model dynamics depending on bottom-up feedforward signal and top-down feedback signal. Without a change of the input signal (e.g., static input) model activities tend to converge after a few iterations. However, when the input signal temporally changes this leads to a conflict between bottom-up and top-down signals. Thus, the system acts like a short-term memory maintaining the actual state for a few time steps. Consistently, if the material appearance changes from opaque to transparent over time one would expect that the perception of the apparent stimulus is more prolonged in time. From this it follows that our model simulations predict a perceptual hysteresis effect for discrimination between opaque-transparent and transparent-opaque transitions induced by a top-down feedback mechanism. Such a hysteresis effects has been already observed psychophysically for motion direction disambiguation (leftward motion vs. rightward motion) [67]. Since both motion and form processing are based on the same neural principles we expect that the predicted hysteresis effect can also be measured psychophysically. Therefore, we are currently planning to investigate experimentally whether our model predictions are validated or not.

### The role of junctions in visual perception

Our core model integrates like-oriented contrasts to simulate the process of contour perception in the visual system. When contours of different orientation meet at the same place non-collinear orientation combinations, namely junctions, are formed. The formation of junctions provides important cues, e.g., for the occurrence of occlusions and transparencies. Occlusions occur in almost every real word scene, and thus, surface completion is a fundamental visual process. In the following, we discuss the individual role of some basic junction types that can be extracted by our model.

#### Transparency

It has been suggested that the perception of transparency is triggered by X-junctions formed by junctions of contours of the transparent and opaque regions at the overlapping area [68]. However, the presence of X-junctions is necessary but not sufficient to elicit a strong transparency effect. In addition, the luminance contrast around the X-junction must follow the two rules: (1) the direction of luminance contrast across an opaque border cannot change in the transparent region; (2) the luminance difference across an opaque border must be reduced in the transparent region [69], [70]. A violation of these rules strongly diminishes the perception of transparency.

Wolfe and collaborators [71] explored in a series of visual search experiments which cues are relevant to guide attention in a search for opaque targets among transparent distracters or vice versa. One of the experiments showed that performance is impaired when X-junctions are removed from transparent items. Another experiment showed that efficient search is still possible if X-junctions are merely occluded (i.e. an occluding bar is used that disrupts the X configurations). In summary, these findings show that indeed X-junctions play an important role in the perception of transparencies, but there seem to be many other factors that play an additional role for transparency perception. Nevertheless, our proposed architecture facilitates the perceptual interpretation of X-junction as proposed by Wolfe and colleagues.

#### Occlusion

When an opaque surface occludes another surface of different luminance a T-junction is formed at the position where the boundary contours intersect each other. If the surface in front has the same luminance than the background the T-junctions collapses to an L-junction. We have shown that our model initially detects such configurations as L-junctions. After a short period of time, when contours are completed over gaps, such L-junctions are recognized by the model as T-junctions.This is consistent with the more context driven interpretation, as observed by Rubin [1]. Rubin investigated in psychophysical experiments how local occlusion cues, such as T-junctions and more global occlusion cues, specifically relatability and surface similarity, play a role in the emergence of amodal surface completion and illusory contour perception. Two contour fragments are *relatable* when they can be connected with a smooth contour without inflection points [71]. Rubin proposes that local T-junction cues can initiate completion processes and that relatability plays a part at later stages.

Interestingly, in rare cases, T-junctions can also support the perception of X-junctions [72]. In their psychophysical experiments, the T-junctions were perceived as having an additional illusory contour leading to the perception of an X-junction (termed “implicit X-junction”). This special case shows that T-junctions do not always lead to the perception of occluding opaque surfaces but can itself be altered in the more global context when prototypical surface patches are formed which may lead to a reinterpretation of local features.

#### Figure-ground segregation

Separating figure from background is one of the most important tasks in vision. Figure and ground information in an image can be represented by assigning ownership of the border between two surfaces. The figure which occludes parts of the background leads to specific boundary configurations, in particular T-junction configurations which can help in the assignment of figure and background. In more detail, the stem of the “T” is formed by the boundary contour of the background surface while the top of the “T” corresponds to the boundary contour of the figure. Motivated by physiological evidence for cells that are selective for border-ownership information [37] some models were proposed where cues signaled by T-junctions are used to generate consistent representations of layered surfaces [30], [38]. This underlines the importance of T-junction cues for the visual system.

#### Motion perception

Junctions do not only play a role in static scenes, they are also important in the context of motion perception. An object's motion cannot be determined from a single local measurement on its contour which is commonly known as the aperture problem [73]. However, at positions where multiple oriented contrasts (i.e. two-dimensional features, such as corners and junctions) are present the ambiguity can be resolved and further be propagated along object contours to get a more global motion percept [74]. Thus, tracking of two-dimensional features over time is a fundamental task in the analysis of motion signals.

**Figure 16:**
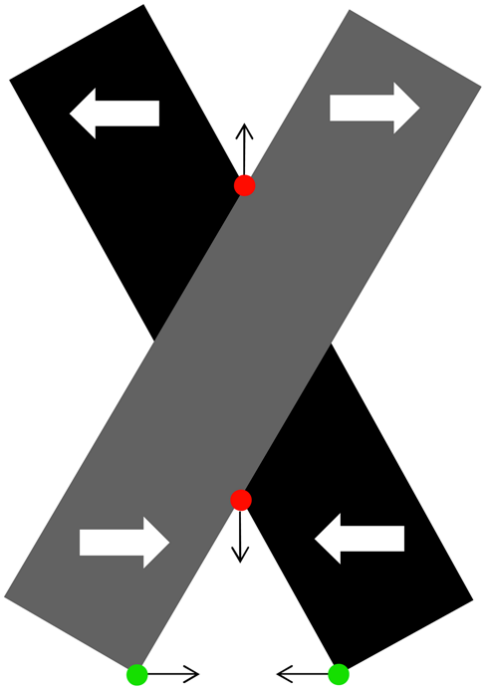
Dynamic stimulus where two occluding bars move in opposite direction. Tracking of L-junctions (green) leads to correct motion estimates of the two bars while tracking of T-junctions leads to erronous motion estimates. Thus, the visual system might use form information, e.g., surface-based occlusion cues to selectively discount local motion estimates for moving T-junctions.

In a study by Pack et al. [75] it is suggested that end-stopped V1 neurons could provide local measures of two-dimensional feature correspondences in motion by responding preferentially to moving line endings. However, the results of Gue et al. [76] contrast with the suggestion that end-stop neurons can determine global motion directions. They propose that lateral and feedback connections play a critical role in V1 motion information integration. But still, it remains unclear whether cortical neurons represent object motion by selectively responding to two-dimensional features such as junctions and corners. On the other hand, motion of specific junction configurations, in particular T- and X-junctions generates erroneous motion trajectories. As shown in Figure 16, if edge motion from two bars moving in opposite horizontal directions is combined, the resulting intersection of constraints is in an incorrect vertical direction [77]. Thus, static form cues such as detected T-junctions could be selectively discounted in the process of motion interpretation [78].

### Limitations of the model

**Figure 17:**
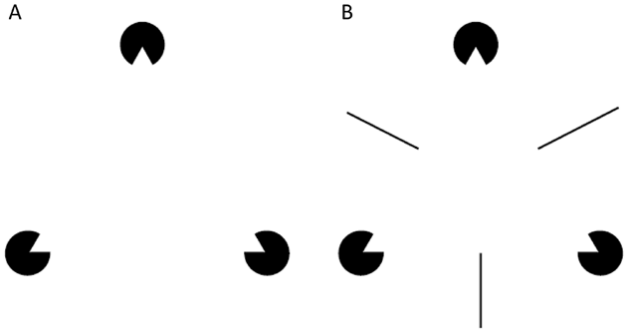
Stimuli where the model fails to produce illusory contour activations. Distances between the starting points of the contours are too large to be bridged by a V2 bipole cell.

Although we have shown that our model is able to produce results that are in line with several empirical findings, there are also some shortcomings of the model. For instance, consider a Kanizsa figure such as illustrated in Figure 17 where the gaps between contour elements are so large that the V2 bipole filter cannot bridge the gap. In this case the model would fail to produce an illusory contour signal in V2. Nevertheless, human observes still have a weak impression of seeing an illusory triangle. We suggest that higher visual areas such as V4 also play a role in illusory contour processing. Evidence comes, e.g., from Pasupathy [79] who found responses to contour features in macaque area V4.

Another restriction of the model is that it does not account for different image scales. Consequently, the model focuses on fine details and suppresses coarse structures. However, the model could be provided with a pyramid of differently scaled version of the same input image. This would correspond to simply replicating the model at multiple scales. An alternative approach would be to employ scaled versions of Gabor wavelet filters in the input stage. Finally, our model does not explain how occlusion-based junctions can be distinguished from texture-based junctions. In this model, we only used stimuli that have homogenous surface reflectance properties. Thus, contours are interpreted as surface borders by the model. In natural scenes, surfaces are often textured due to surface material properties which would also lead to junctions signals and thus to ambiguities in the interpretation. However, such ambiguities could be solved in higher visual area such as V4 [80] which are not in the scope of this paper. In addition, stereo information can also help to correctly identify occlusion-based junctions.

**Table 1:** Modulatory effect of feedback (FB) signal on feedforward (FF) signal.

FF	FB	new activity after FF–FB combination	modulatory effect
ON	ON	∝ FF⋅(1+FB)	+
ON	OFF	= FF	0
OFF	ON	0	0
OFF	OFF	0	0

‘ON’ means activity present and ‘OFF’ means no activity present. Modulation (**+**) of feedforward activity takes only place when both signals, feedback and feedforward are present (ON). Otherwise, feedforward activity remains unchanged (**0**).

## Supporting Information

Appendix S1 Model equations describing the processing at individual model stages.
(0.22 MB DOC)
