# Supplementary material for: Extraction of Surface-Related Features in a Recurrent Model of V1-V2 Interactions
Source: PLoS One. 2009 Jun 15;4(6):e5909. doi: 10.1371/journal.pone.0005909 (PMC2691604; doi:10.1371/journal.pone.0005909)
Supplement: Appendix S1 — Model equations describing the processing at individual model stages. (0.22 MB DOC) [file pone.0005909.s001.doc]

# Appendix S1

In the following, the model equations describing the processing at individual model stages are depicted.

## V1 simple and complex cells (lower model area V1)

To extract basic form features, such as discontinuities in the luminance distribution, we use Gabor wavelet responses which were computed from the input signal. In the initial processing stage, which corresponds to lower *model area V1*, we use a distributed representation of N filter orientations between 0° and 180° at each spatial position. We use a DC-compensated version of Gabor filters [81] which is defined in the following equation:

(1)

Here, $\vec{x}$ is a 2D-vector of spatial coordinates and is the wave vector that defines the wave length and points in direction . Parameter$\sigma$ ($\sigma$=2.7) defines the width of the Gaussian envelope function. The image intensities are filtered with the orientation selective complex filter kernels leading to N maps, denoted by :

. (2)

The impulse responses respectively correspond to receptive filed properties of *simple cells* that have a cosine wave (real part) and a sine wave (imaginary part) restricted by a Gaussian envelope function.

As we do not make use of local phase or polarity we combine information by calculating the absolute value of (full-wave rectification), such that

. (3)

The signal corresponds to activity of complex cells and is interpreted as initial feedforward estimate of local oriented structure in lower model area V1.

## Normalization of complex cell responses (centre-surround inhibition)

Local variations in illumination or surface reflectance can considerably influence the local luminance contrast level. Thus, it is important to locally normalize for contrast. We employ a non-linear inhibition mechanism that is based on a combination of divisive and subtractive centre-surround interactions. Importantly, this operation leads to an interaction between cells selective for different orientations. Thus, locally ambiguous contour signals represented by evenly distributed orientation responses are weakened by this process while locally unambiguous contour signals as represented by a unimodal distribution over the orientation space are left unchanged. Furthermore, activity of cells in a spatial surround can also inhibit activity in the centre to a certain degree. Divisive inhibition is also known as *shunting* inhibition[18]. Centre-surround inhibition is applied at several stages of the model. Therefore, we define an inhibition operator which equates in

. (4)

Inhibition of complex cell responses is performed by applying the inhibition operator to complex cell responses

. (5)

$G_{\sigma}$ ($\sigma$=2.5) denotes an isotropic 2D-Gaussian kernel that defines the local surround where cell activities are pooled to have an inhibitory effect on the centre. Parameter γ=2.5 is used to control the amount of subtractive inhibition, parameter β=1 controls the nonlinear saturation characteristics and parameter α=1 is used to control the range of the output values. Note that output values are half-wave rectified in order to avoid negative output (denoted by [.]^+^).

## V1 bipole and end-stop cells (upper model area V1)

Bipole cells and end-stop cells both receive input from normalized complex cell responses .

Bipole cells are used to model long-range lateral connections in area V1. We used two additively connected anisotropic Gaussian kernels to model the filter:

(6)

In order to generate rotated versions of the bipole filter we rotate the coordinate system in the appropriate direction.

(7)

The ratio between the two principal components of the anisotropic Gaussian is defined by vector $\vec{k}$. Furthermore the two anisotropic Gaussian kernels are displaced by the amount of $\delta$ apart from each other along the first principle axis.

End-stop cells are used to model end-inhibition properties of V1 cells. We used an isotropic Gaussian to model the inhibitory part as well as an elongated Gaussian to model the excitatory counter-part. Since end-stop RFs are not symmetric, two mirror-symmetric versions are modeled for each orientation, denoted by $E^{0}$ and $E^{180}$.

(8)

(9)

Parameter $\eta$ is used to adjust the strength of the inhibitory filter part. Vector $\vec{k}_{e}$ defines the elongation properties of the excitatory Gaussian sub‑kernel. Parameters $\delta_{e}$ and $\delta_{i}$ are used to displace the excitatory and the inhibitory filter part such that the centre of the filter where activity is integrated lies approximately in‑between the two Gaussian sub‑kernels. Note that filter responses cannot reach negative values as we use half-wave rectification.

In the following operation the activity of lower area V1 from normalized complex cells is filtered (convoluted) with populations of end-stop cells and bipole cells.

(10)

(11)

Afterwards, the resulting activities of perpendicular oriented bipole and end-stop cells are additively combined. Finally centre-surround inhibition of cell activity is performed at the output of model area V1.

(12)

## V2 bipole cells

Mode V2 receives input activity from model area V1. According to physiological evidence our model V2 cells have larger receptive fields than V1 cells. Moreover, the responses properties of bipole cells in V2 suggest for an AND-like connection of the two sub‑fields. Accordingly, V2 bipole filters are realized by a combination of multiplicatively connected anisotropic Gaussians.

(13)

This operation has the effect that activity that is integrated from one of the two sub‑fields is not sufficient to produce activity at the output of the filter stage.

The output of model area V2 is further computed by filtering input activity with V2 bipole filters .

(14)

Finally, the result of the convolution undergoes a center-surround inhibition stage.

(15)

## Recurrent V2-V1 feedback

All described mechanisms so far are based on feedforward computations. Feedback signals can be used to transfer information that is present at higher stages (V2) of the cortical hierarchy to lower stages (V1) where information about contours is more incomplete or ambiguous.

Here, we recurrently fed back output activity from model area V2 to modulate initial activity of model V1 complex cells.

(16)

The feedforward signal acts as a driver while the top-down feedback signal is a modulator. The driving signal gates the feedback signal (Table 1). According to the multiplicative connection the feedback signal cannot generate new activity at positions where feedforward activity is zero. In addition, zero feedback cannot weaken initial activity which is in accordance with physiological evidence that feedback has mainly driving character. Only in the case when both, feedforeward and feedback signals are present, the feedforward signal is modulated. Parameter ω in Eq. 16 is used to adjust the strength of the modulation.

In our mode, feedforward and feedback computations are performed alternately until model responses converge to a stable state.

## Extraction of junctions signals

Junction signals are extracted and combined from activities of cell populations from model V1 end‑stop cells and V2 bipole cells. The read-out process of T-, X-, and L-junction signals is described by the following equations.

(17)

(18)

(19)

For each of the three signals, activities from pairs of cells are multiplicatively combined, such that both cells have to be active in order to generate a junction signal. This is done for all pairs of orientations except pairs of cells corresponding to same orientations (see Figure 6).

For each junction type, the results of all pairwise combinations are integrated to obtain junction signals that are independent of junction orientation.

## Interaction between junction signals

T-, L-, and X-Junction signals can locally compete with each other through centre-surround inhibition to prevent multiple junction types being signaled in a small local surround. Moreover, junction signals are normalized such that they have the same range of values. The mechanisms described in the following equations are similar to those employed in the recurrent model computations.

$S^{X}=\frac{\left[S^{X}-\alpha\left(S^{T}*G_{\sigma}\right)-\beta\left(S^{L}*G_{\sigma}\right)\right]^{+}}{\gamma+\left(S^{X}*G_{\sigma}\right)}$ (20)

$S^{L}=\frac{\left[S^{L}-\alpha\left(S^{X}*G_{\sigma}\right)-\beta\left(S^{T}*G_{\sigma}\right)\right]^{+}}{\gamma+\left(S^{L}*G_{\sigma}\right)}$ (21)

$S^{T}=\frac{\left[S^{T}-\alpha\left(S^{X}*G_{\sigma}\right)-\beta\left(S^{L}*G_{\sigma}\right)\right]^{+}}{\gamma+\left(S^{T}*G_{\sigma}\right)}$ (22)

Parameters α and β adjust the inhibitory influence of junctions signals in the competition process. Parameter gamma regulates the influence of divisive self-inhibition that is performed.
